# 

**Published:** 1881-10

**Authors:** 


					﻿OCTOBER, 1881.
TWENTY-EIGHTH YEAR.
HALL'S
JOURNAL
OF
j__ «	SHP—	__ |
’HEALTH'
E. H. GIBBS, A.M., M.D., Editor,
No. 141 EIGHTH STREET (Near Broadway), NEW YORK.
TERMS: $2 a Year, or $1 for Six Months, Single mler, 20 cts. <
				

